# Co-production of a youth advocacy video on the harms of e-cigarette advertising in Scotland

**DOI:** 10.1093/heapro/daae097

**Published:** 2025-03-05

**Authors:** Marissa J Smith, Caroline Vaczy, Shona Hilton

**Affiliations:** MRC/CSO Social and Public Health Sciences Unit, University of Glasgow, Glasgow G3 7HR, United Kingdom; MRC/CSO Social and Public Health Sciences Unit, University of Glasgow, Glasgow G3 7HR, United Kingdom; Department of Social Work and Social Policy, University of Strathclyde, Glasgow G4 0LT, United Kingdom

**Keywords:** advertising, determinants of health, participatory, photography, qualitative methods, adolescent

## Abstract

This study aimed to investigate young people’s exposure to and perceptions of e-cigarette advertising to co-produce an advocacy video. This focus on e-cigarette marketing and its targeted appeal to young people comes at a crucial juncture, as policymakers in the UK and Scotland channel considerable efforts into shaping new regulations in response to these concerns, such as banning disposable e-cigarettes. The research to co-design a video was conducted with 33 young people aged between 12 and 16 living in the Central Belt of Scotland. The research comprised four stages: workshops, photo elicitation, focus groups and video development. Young people expressed concerns regarding the potential health effects of e-cigarettes, the ubiquity of e-cigarette advertising and products seemingly directed at young people, and the use of e-cigarettes among their peers. While none of our participants identified themselves as e-cigarette users, and all were below the age of 18, some mentioned seeing targeted advertisements for e-cigarettes online. These concerns were also reflected in participants’ contributions to the video production process. Our findings highlight that young people feel overly exposed to e-cigarette advertising and they identified aspects of these adverts (including the use of vibrant colours and flavour variations) that they felt were designed to appeal specifically to young people. These findings suggest the need for stronger legislation to protect young people from the advertising and marketing of e-cigarettes. Further research might also usefully contribute to understanding counterarguments and marketing from public health advocates to limit the appeal of e-cigarettes to young people.

**Contribution to Health Promotion**

- This timely research on e-cigarette advertising is crucial amidst current efforts of UK policymakers to enact more stringent regulations.
- Co-production methods offered original insights into the issues most concerning to young people (e.g. over-exposure and design of e-cigarette adverts), and counter representations using engaging media such as animated video to increase young people’s awareness of the issues associated with e-cigarette advertising.
- Our findings suggest the need for stronger legislation to curb e-cigarette advertising and marketing, safeguarding young people from targeted marketing of products they are prohibited from purchasing.

## BACKGROUND

Concerns have been raised by researchers, advocacy workers and young people themselves over the uptake of e-cigarettes (‘vapes’) among young people (Action on Smoking and Health, 2023). In Great Britain (GB), the proportion of young people (aged 11–17) who have experimented with e-cigarettes has grown significantly in the past year (up from 7.7% in 2022 to 11.6% in 2023) (Action on Smoking and Health, 2023). Despite varying opinions within the public health community about e-cigarettes’ value in harm reduction for adults and uncertainties regarding their long-term health effects, there’s widespread agreement on the importance of preventing young people from initiating vaping (Hilton *et al*., 2016; Khan, 2022; McNeill *et al*., 2022).

Each year, the tobacco industry spends tens of billions of dollars to market its products (World Health Organization, 2013) and uses increasingly sophisticated and covert forms of tobacco advertising, promotion and sponsorship (TAPS) (Tapera *et al*., 2021). The rationale behind Article 13 of the WHO Framework Convention on Tobacco Control (FCTC) (World Health Organization, 2003) and its implementation guidelines is to offer a course of action against TAPS by the tobacco industry. Before plain packaging regulations, tobacco companies used colourful packaging to make smoking more appealing (Wayne and Connolly, 2002; Anderson *et al*., 2005; Carpenter *et al*., 2005). This effective marketing strategy, among others used previously to market cigarettes, has now been adopted by e-cigarette manufacturers (Campaign for Tobacco-Free Kids, 2013; Walley *et al*., 2019; Haugh, 2020). Research suggests that the bright packaging, eye-catching designs and variety of flavours can attract young people to try these products (Kong *et al*., 2015; Audrain-McGovern *et al*., 2016; Czoli *et al*., 2016; Zare *et al*., 2018; Okawa *et al*., 2020; Kalaji, 2022). Marketing strategies used by e-cigarette companies have been associated with the uptick in e-cigarette use among young people (Hammond *et al*., 2020). These concerns have led to studies on e-cigarettes across various media platforms, including traditional (TV, print, outdoor) and social media (websites, social networks, blogs). The European Union (EU) Tobacco Products Directive 2014/40/EU (TPD) (European Commission, 2014), introduced in 2016 and transposed into UK law through the Tobacco and Related Products Regulations 2016 (TRPR) (UK Government, 2016), set rules on how e-cigarettes can be advertised. The TPD (UK Government, 2016) prohibited the advertising of nicotine-containing e-cigarettes (unless licensed as medicines) in channels with potential cross-border impact (i.e. channels that show adverts or sponsored events that originate from non-EU countries in EU countries), including TV, radio, newspapers, magazines and sponsorship (Stead *et al*., 2019; Smith and Hilton, 2023). Additionally, the UK Advertising Standards Authority (ASA) Committee of Advertising Practice (CAP) Code and Broadcast Committee of Advertising Practice (BCAP) Code, updated in 2017, further govern e-cigarette advertising, prohibiting TV and radio ads; however, billboards and posters in shops are not included in the scope of the TRPR and are currently permitted but allowing billboards and shop posters (ASA, 2019a, 2019b).

E-cigarettes are promoted creatively through social media, with well-designed features including colours, flavour variations, incentives (such as price promotions and discount vouchers) and even celebrity endorsements (Zhu *et al*., 2014). Previous studies which examined e-cigarette-related social media (Cole-Lewis *et al*., 2015; Johnson *et al*., 2017; Chen *et al*., 2020; Ketonen and Malik, 2020; Scheffels *et al*., 2023; Smith *et al*., 2023) found that the vast majority of the content depicted positive attitudes towards vaping, while negative characterizations were mostly absent. Online e-cigarette advertising, even when disguised as peer recommendations, can increase the likelihood of vaping, especially among young people and non-smokers (Phua *et al*., 2017; Amin *et al*., 2020).

Considering the rapid growth and popularity of e-cigarettes and near absence of regulation of e-cigarette advertising, this research aimed to investigate young people’s exposure to and perceptions of e-cigarette advertising to co-produce an advocacy video.

## METHODS

This original research followed a multi-stage, multi-method approach consisting of workshops, photo elicitation, focus groups and video development. Our aim when selecting this methodology was to build rapport with participants, diversify the types of data gathered and deepen our understanding of the young people’s views with each subsequent phase. Given the creative nature of co-creating a video, we sought to allow participants to engage with the topic in creative ways throughout the project. The use of co-production and multiple modes of data collection facilitated active involvement and collaboration between researchers and participants. In this section, we will discuss the method of co-production and recruitment of the young participants before describing the four stages in more detail.

We aimed to use co-production methods where researchers, practitioners, the public and young people collaborate to share power and responsibility (Hickey *et al*., 2018). By working with a video production company, we drew on each group’s expertise to produce an effective video. Co-producing research with young people ensures their voices are central to the project, resulting in research that is richer, more relevant and better tailored to their needs (Kesby, 2000; Smith *et al*., 2002; Grover, 2004; Thomas, 2007).

### Sampling and recruitment of young people

We purposively sampled young people aged 11–16 from the Central belt of Scotland, representing a diverse sample in terms of sex and socioeconomic background. Groups of participants were recruited through local youth organizations and schools, facilitated by youth workers who distributed information sheets and maintained communication with researchers. One of the original 33 participants did not upload photos for Stage 2 of the project, and scheduling difficulties with one youth group led to the loss of five participants between Stage 2 and 3 of the project.

Invitees who agreed to participate received a participant information sheet, privacy notice and consent form. Prior to the workshops, participants completed a short questionnaire about their age, sex, postcode and past/current use of traditional cigarettes and e-cigarettes at the time of the study.

### Stage 1: workshops

Conducting workshops allowed for openness through the presence of group members of a similar age or from the same organization (e.g. youth group) but avoided the potentially intimidating aspects of a larger group discussion. The workshops aimed to introduce the research project and its objectives, engage participants in activities related to e-cigarette advertising experiences and generate ideas for the video.

Three workshops held from March to April 2023 involved 33 participants (10–12 per workshop). Structured activities focused on e-cigarette advertising and video development (Supplementary Appendix A). Workshops were moderated by three researchers who posed questions and encouraged participants to explain their thoughts and opinions in more detail. Workshops lasted about 2 hours at school or youth organization sites. While not recorded, researchers took field notes, and materials produced were kept for analysis. Each participant received a £20 shopping voucher as compensation.

### Stage 2: photo elicitation

Participant-generated photographs are widely used in qualitative research, including studies exploring young people’s experiences of social processes and settings (Croghan *et al*., 2008; Barker and Smith, 2012; Zartler and Richter, 2014; Shirani *et al*., 2016; Neale, 2017), as they are an accessible method for young participants. In this project phase, participants were asked to capture photos or short videos of e-cigarette advertising encountered in their daily lives, including on social media. Privacy considerations led participants to avoid photographing individuals, but advertisements featuring people were permissible. These images served as discussion prompts in subsequent focus groups and informed the video storyboard development. Photographs from 32 participants were uploaded to a secure file-sharing website hosted by the University of Glasgow. Upon receipt of the photos, participants were given a £20 voucher as compensation for their time. Instead of analysing the content of photos or videos, our approach focused on the narratives and descriptions provided by the participants themselves, facilitating a deeper understanding of their experiences and perspectives during Stage 3 of the project.

### Stage 3: focus groups

Six focus groups were conducted between April and May 2022. Focus groups included between 3 and 6 participants (a total of 28 participants), allowing for an in-depth and interactive exploration of participants’ opinions and experiences with e-cigarette advertising. Each participant was given a £20 shopping voucher as compensation for their time.

Focus groups used a slideshow of Stage 2 photos and a topic guide. Discussions were led by one or two researchers, promoting participation and depth. Four online sessions used Microsoft Teams, and two were in-person at the youth organization. Groups lasted 48–75 minutes and were audio recorded with consent. Researchers made brief notes, with one researcher listening to the audio-recording to cross-compare and make more detailed notes from recordings.

Focus group transcripts were thematically analysed following Braun and Clarke’s six-phase framework for thematic analysis (Braun and Clarke, 2022). Thematic analysis was chosen for its strength in highlighting similarities and differences between participants’ statements and allowing for the emergence of unanticipated insights (King, 2004; Braun and Clarke, 2022, 2024). The research team read and reread the transcripts to become familiar with the data, and then iteratively constructed a coding frame (Supplementary Appendix B) developed by two members of the research team, structured into themes based on the research questions, with additional themes drawn inductively from the data. Each transcript was imported into NVivo V.12, coded independently, cross-checked and analysed by M.J.S. and C.V. Contradictory cases and group dynamics were discussed, making use of transcripts and field notes.

Quotations from focus group discussions, condensed for brevity without compromising meaning, are reproduced in the results section to illustrate analysis points while ensuring participant anonymity.

### Stage 4: video production

In the project’s final stage, the research team collaborated with a video production company, Media Co-Op, and young people to create a video that captured their ideas. Through iterative co-creation, the video aimed to convey important messages using eye-catching visuals. Media Co-Op led workshops with the research team to understand the video’s objectives, the e-cigarette advertising landscape and the young people’s perspectives gathered in earlier stages. They also designed materials to better understand the young people’s vision and preferences for the video. Participants provided input on video styles, with a subgroup of four young people offering feedback on specific stylistic elements. Regular feedback with the research team guided production, and final voiceovers were recorded by two young people to maintain inclusivity and appeal to the target audience.

Ethical approval for this study was granted by the College of Medical Veterinary and Life Sciences Research Ethics Committee at the University of Glasgow (reference 200220088).

## RESULTS

The results are presented by project stage, beginning with the workshops, followed by photo elicitation and focus group discussions. Production of the advocacy video is detailed in Supplementary Appendix C.

### Stage 1: workshops

Thirty-three young people aged 12–16 participated in this stage of the study [20 females (61%) and 13 males (39%)]. This sample represented a wide diversity in sociodemographic characteristics, based on postcodes collected in pre-workshop questionnaires on demographic information and experiences with e-cigarettes. The age distribution within the sample was weighted slightly more heavily towards 14–15-year-olds, with 14-year-olds making up the largest subgroup (*n* = 14). See Supplementary Appendix D for workshop composition

The workshop findings are categorized under four headings: perceived prevalence and design of e-cigarette adverts; perceived health impacts of e-cigarettes; alternative advertising strategies; and video styles. Illustrative quotations are presented in this section; however, due to the dynamic of the workshops, we are not able to attribute quotes to specific participants.

#### Perceived prevalence and influence of e-cigarette adverts

Participants described seeing e-cigarette adverts daily, including on social media platforms, in shops, at transport facilities (e.g. bus stops, train stations). One participant stated, ‘I see them every time I go out the house’ (Workshop 3). When asked ‘do e-cigarettes adverts influence your choice to use these products’, several participants answered yes, for example, ‘They [adverts] make me what to try it and see what the flavours are like’ (Workshop 2).

Several participants stated that their behaviour was not influenced by e-cigarette adverts but acknowledged that advert design makes the products enticing.

The adverts don’t make me want to use them but the colours and flavours they do draw my attention. (Workshop 2)

Interestingly, participants often spoke about their friends using e-cigarettes, which they found to be more influential than e-cigarette adverts.

Seeing others using them like my friends tempts me to use them. (Workshop 1)

#### Perceived health impacts of e-cigarettes

In an effort not to influence the young people’s ideas, we asked them to consider ‘what are the potential health impacts of e-cigarettes?’ Participants predominately referred to potential negative health impacts like gum disease, popcorn lung, lung cancer and addiction, with some expressing concern about unknown long-term health effects.

It affects your lungs and gums, but we don’t know the long-term effects because they’ve not been around long enough. (Workshop 3)

One participant stated that they were unaware of any health impacts.

I don’t think it [vaping] does anything to your health but it could have long-term damage, but we don’t know. (Workshop 1)

Participants did not discuss the potential health impacts of e-cigarette use among people who smoke and use them to quit or reduce harm. Participants often stated that the potential negative health impacts would discourage them from using e-cigarettes, however, some expressed a tension between the fear of health consequences and the draw of the products.

The health effects are too bad, but the colours and flavours are appealing. (Workshop 2)

#### Views on marketing and advertising of e-cigarettes

Many participants advocated for policies to restrict adverting of e-cigarettes, with several discussing the impact that this would have for young people.

[Introducing policies] would make young people less likely to buy them because they wouldn’t be able to see advertising or talk about the adverts they see as. (Workshop 3)

Other participants stated that introducing restrictions would not solve the problem as young people would still find a way to purchase e-cigarettes, with one pointing out the strong effects of peer influence.

I think not much would change [if policies were introduced] as kids don’t find out about vapes through adverts, they mostly find out about them through their friends. (Workshop 2)

Several participants suggested that e-cigarette advertising should not be banned but that additional regulations should be placed on it to make the products less appealing to children and young people.

I think they should be advertised but not with bright colours. (Workshop 1)

### Stage 2: photo elicitation

Of the 33 initial participants, 32 submitted photos for Stage 2. The aim of this stage was for young people to capture photos and/or short videos of e-cigarette advertising they encounter in their daily lives. We did not analyse the content of photos or videos but rather used them as discussion prompts during the focus group discussions in Stage 3 of the project.

### Stage 3: focus groups

Of the 33 initial participants, 28 young people aged 12–16 [16 females (57%) and 12 males (43%)] participated in this stage of the study. The age distribution within the sample was skewed slightly towards 14–15-year-olds, with 14-year-olds making up the largest subgroup (*n* = 12). See Supplementary Appendix E for focus group composition.

#### Views on youth exposure to e-cigarette advertising

**Fig. 1:**
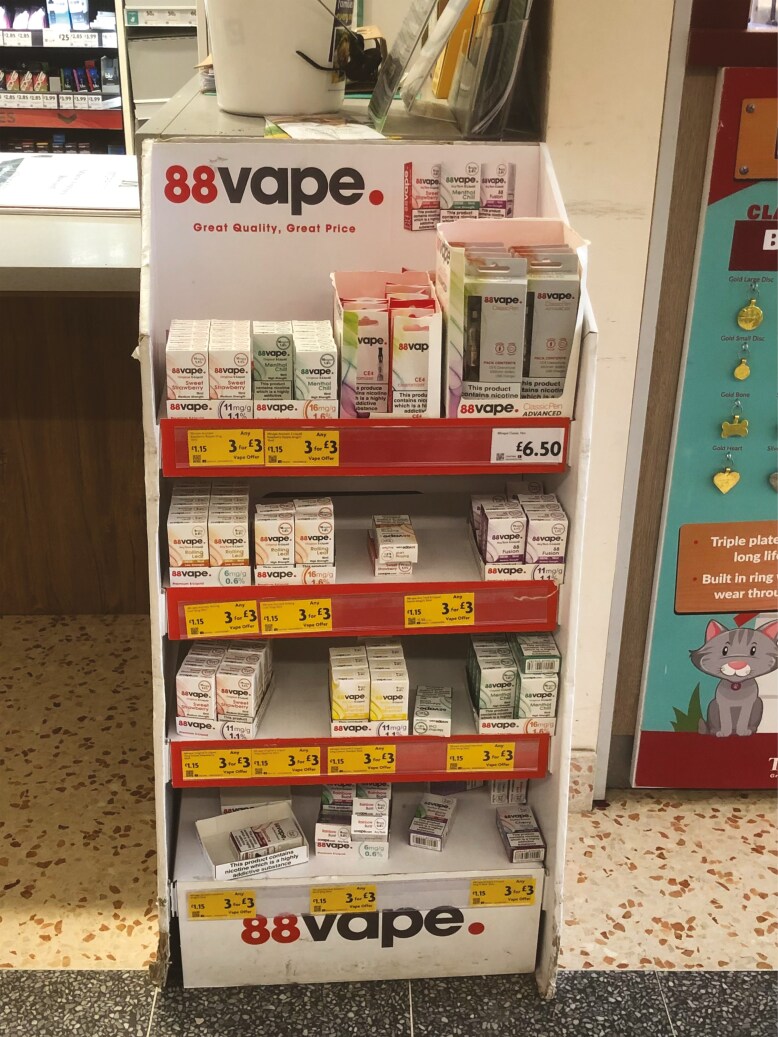
88 Vape e-cigarette display near to a till.

Young people discussed frequent exposure to e-cigarette adverts in numerous locations, including supermarkets where posters were visible both inside and outside of shops. They noted adverts in corner shops were strategically placed at the tills to entice people to try the products. Participants also discussed the convenient location of many of these shops near schools, facilitating young people’s exposure (Figure 1).

After vapes became such a big trend, the corner shop introduced advertising on the counter where you pay […] it’s just a wall full of vapes. […]. The majority of teenagers in the school, they use that corner shop. The colours they use, the deals and say this flavour is new, it’s great, just try it, [it influences] people, especially teenagers. (Female, 16, never smoker, never vaper)

**Fig. 2:**
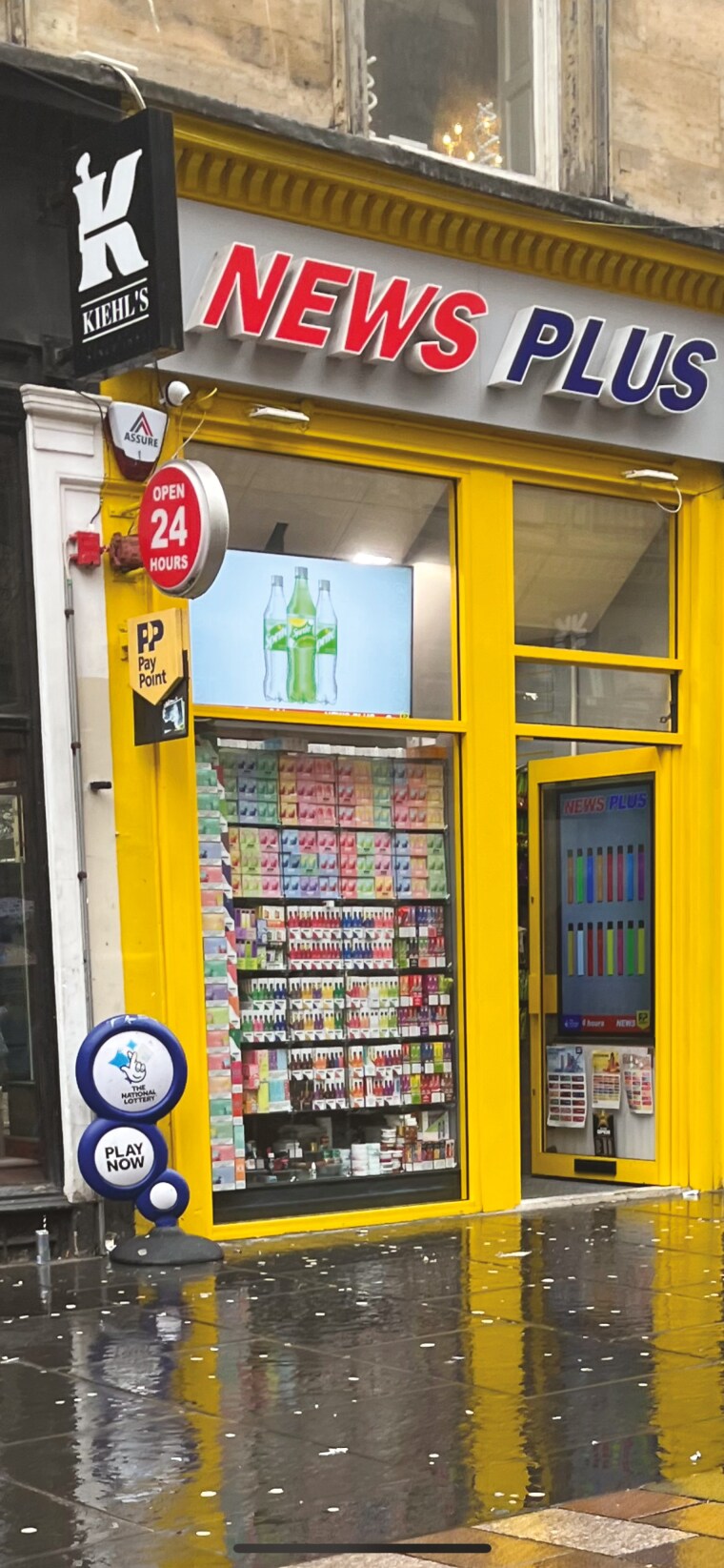
E-cigarettes being displayed in a shop window.

Participants discussed the e-cigarette packages themselves being used to create displays which in turn act as unavoidable adverts (Figure 2).

It’s actually onto the street. It’s a whole lot of bright-coloured vapes, they catch your eyes. You have to walk past it. (Male, 14, never smoker, never vaper)

Although most participants did not report seeing many e-cigarette advertisements on social media, they did discuss social media users (e.g. friends or influencers) discreetly featuring e-cigarettes in their social media posts.

I don’t see anybody saying, oh go and buy this vape, it’s really subtle. If I did see one sitting in someone’s video, I’d be like, oh wait, what’s that, and then once you figure it out, you’re like, oh that’s what that is. (Female, 14, tried smoking, tried vaping)

Participants stated that if they viewed influencers advertising e-cigarettes, it would make them more likely to buy the product, demonstrating the power of trust and loyalty in advertising.

It makes you feel oh, they’re doing it, so it must be fine for me to do it or oh, I wanna do something that they’re also doing, because it feels like you can relate to them. (Female, 14, never smoker, tried vaping)

#### Views on the design of e-cigarette adverts

All participants highlighted the design of e-cigarette adverts, stating that noting the frequent use of vibrant colours that that attracted their attention.

It’s mostly bright colours, the way they design their posters or displays, it makes you want to stop and take a look at them. Even if you’re not interested in buying a vape, it attracts your eyes. (Female, 16, never smoker, never vaper)

Participants highlighted the use of colour in e-cigarette adverts, often used to demonstrate the variety of flavourings of the product available.

All the adverts are all bright and colourful and all different colours and flavours. So, it just draws your attention to it to see what the flavours are. (Female, 15, never smoker, tried vaping)

**Fig. 3:**
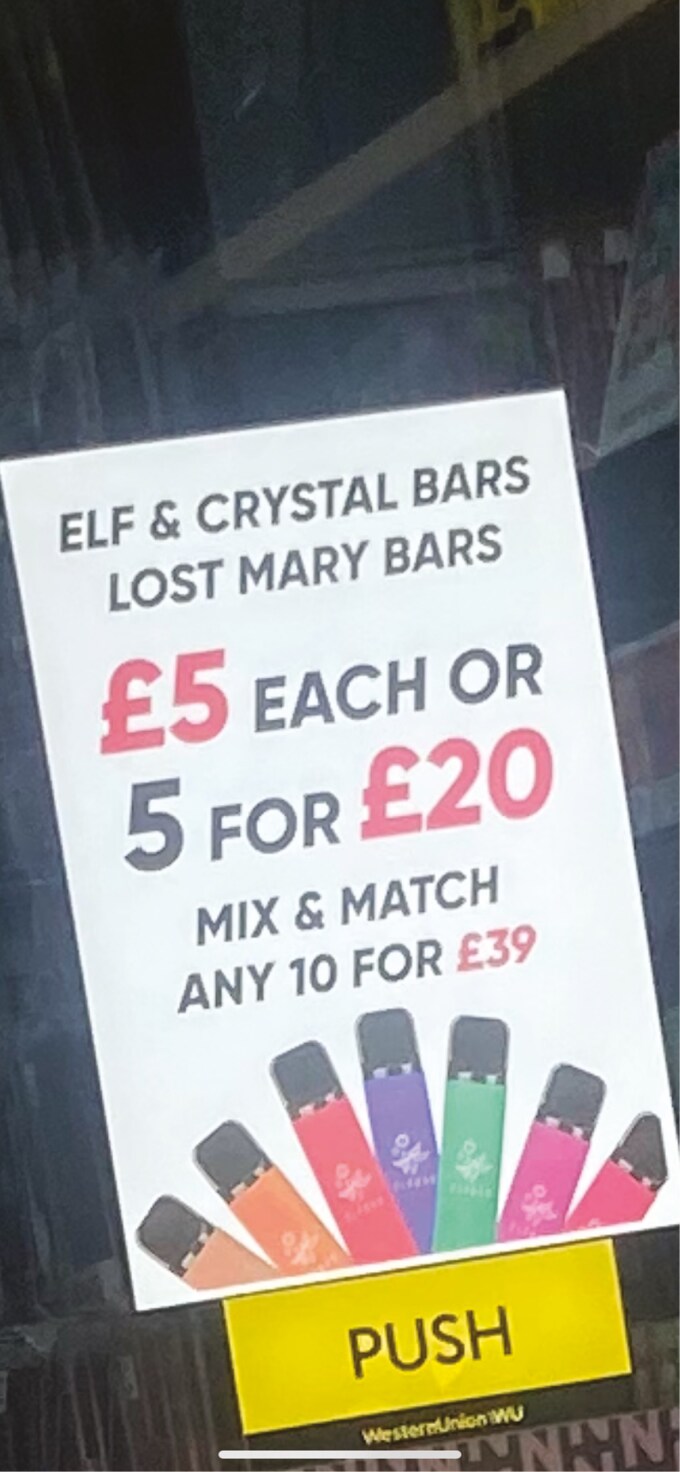
Advert with brightly coloured e-cigarette flavour options and large colourful writing.

Several participants highlighted that specific colours, such as red, were ‘more attractive’ and were often used on promotions, special offers and deals (Figure 3).

The £5 and the £20 is in red writing that would catch your eye. The black writing I don’t think would. (Female, 15, never smoker, never vaper)

**Fig. 4:**
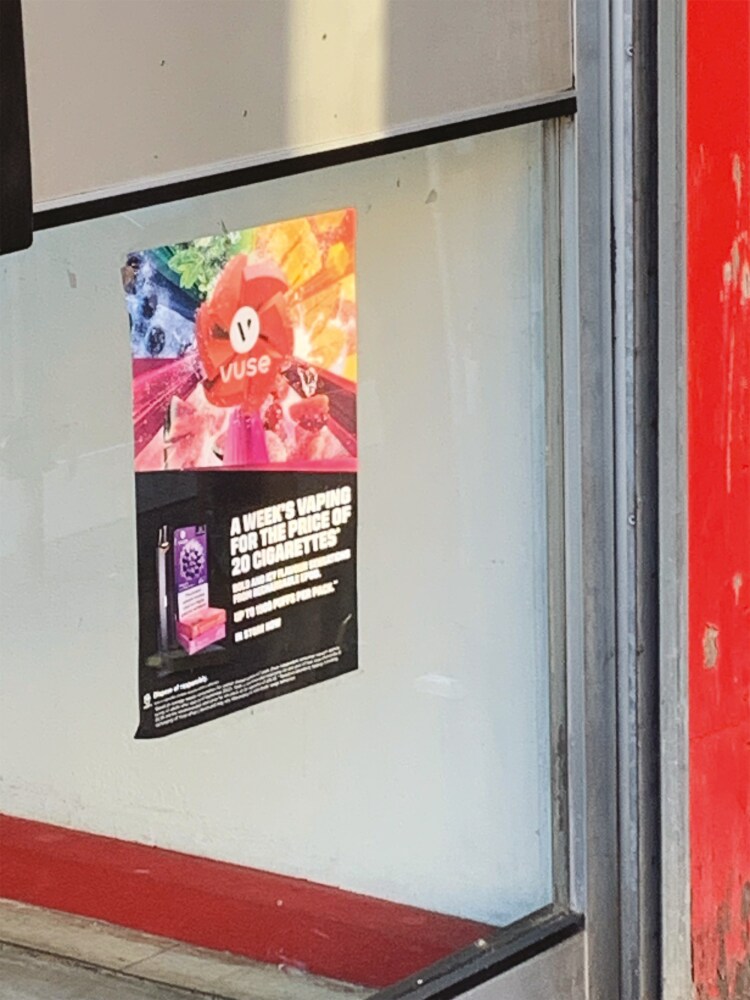
Photo of colourful e-cigarette advert.

One participant highlighted that they were uncertain what was being advertised due to the design of the advert and thought it was confectionery (Figure 4).

“The most striking was the last one ‘cause of the colours of it. […] I thought it was coffee or something because it looks appealing. It kind of looks like candy to me.” (Female, 14, never smoker, tried vaping)

Participants recognized that e-cigarettes seem to be targeted specifically at people their age.

I think they [e-cigarettes] tend to be advertised towards teenagers and kids and because they all use bright colours and they all stand out. If you’re walking down the street they stick out especially because they’re all fruit as well. (Female, 15, never smoker, tried vaping)

Participants discussed how memorable adverts can influence people’s choices, including those of their friends.

I know it influenced my friend, because she said, oh I don’t want to try vapes, then she seen an advert so tried it. (Female, 12, never smoker, never vaper)

#### Perceptions of how e-cigarette advertising could be changed

Participants had divergent views on the future of e-cigarette advertising. Several participants stated that advertising of e-cigarettes should not be allowed as it encourages young people to try e-cigarettes.

Teenagers or young people, they’re the ones that are doing it mostly. So, if they’re advertised, they’re just more likely go into the shop and get one. (Female, 15, never smoker, never vaper)

Other participants thought that the advertising of e-cigarettes should be permitted but changed to appeal less to young people who have never smoked cigarettes.

They could limit the flavours or stop advertising all the different flavours, because there are too many flavours that young people enjoy, and that’s what they like. (Female, 14, never smoker, tried vaping)

The video was launched on 12th December 2023 in a webinar and can be accessed at https://www.youtube.com/watch?v=xwZ-aoQIwjs&t=2s.

## DISCUSSION

This study aimed to investigate young people’s exposure to and perceptions of e-cigarette advertising to co-produce an advocacy video, intended to make a timely contribution to calls to reduce young people’s exposure to e-cigarette advertising. Our qualitative thematic analysis revealed that young people have concerns regarding the potential health effects of e-cigarettes, the ubiquity of e-cigarette advertising and products seemingly directed at young people, and the use of e-cigarettes among their peers. While none of our participants identified themselves as current e-cigarette users, some had been exposed to targeted advertisements for e-cigarettes online, despite being under 18, and many had seen e-cigarettes on social media through ‘subtle’ placement. Participants described targeted e-cigarette adverts mainly from social media influencers, peers and public figures. These experiences speak to the changing advertising landscape and how e-cigarette companies can take advantage of less formal, traditional means of marketing to leverage the social influence of young people and online personalities without breaking the law.

Previous research (Parnham *et al*., 2023) has found that the likelihood of adolescents in Great Britain seeing e-cigarettes increased in the period from 2019 to 2022, while the likelihood of seeing tobacco cigarettes on display decreased. In the UK, as only tobacco cigarettes are covered by point-of-sale (POS) display bans. The rapid rise of disposable e-cigarette use among young people in the UK, driven in part by their low cost and advertising, may have resulted in this increased exposure to e-cigarette products (Tattan-Birch *et al*., 2023). Exposure to e-cigarette displays has been associated with e-cigarette use in Scottish young people and it is possible noticing e-cigarette displays could impact e-cigarette uptake (Best *et al*., 2016). Although our study did not analyse the association between exposure and e-cigarette use, participants in this study did discuss the prevalence and ubiquity of e-cigarette adverts as well as a perception that they were directly targeted at young people. Small shops and corner shops may be important as a source of e-cigarette POS exposure as they are frequently visited by young people. Young people in our study highlighted the prevalence of e-cigarettes adverts and displays in corner shops. The presence of e-cigarettes in corner shops also indicates accessibility which has been shown to influence intention to smoke (Doubeni *et al*., 2008). More recently, there has been increased involvement of the traditional tobacco industry in the e-cigarette market (Cornish *et al*., 2021) and increased marketing of e-cigarette products, in particular online and on social media (Stead *et al*., 2021; Smith and Hilton, 2023; Smith *et al*., 2023). Echoing the results of previous research (Alpert *et al*., 2019; Struik *et al*., 2020; Kostygina *et al*., 2022; Smith and Hilton, 2023), several participants in our study discussed seeing targeted advertisements for e-cigarettes online. The e-cigarette industry is promoting and advertising e-cigarettes to underage populations through influencer and celebrity endorsements, offering a variety of enticing flavours, and using promotional endorsements. Thus, e-cigarette advertising may be influencing young people from two sources—the physical spaces in which they live and the online spaces where they increasingly spend much of their free time.

As with all research, our study has some limitations. Participants’ perspectives may reflect the UK’s unique e-cigarette policies and sociocultural context, potentially limiting generalization. We focused on Scottish youths and this may restrict broader UK applicability, though diverse sampling ensured that the research sample included a diverse range of Scottish young people in terms of age, sex and socioeconomic background. Our recruitment strategy resulted in some groups being weighted towards certain demographic characteristics; for example, one group comprised predominantly of females whilst another group contained mostly young people from deprived areas. However, we did not find any differences in experiences by these characteristics. Varied data collection methods (online versus face-to-face) and settings (classrooms, youth centres) may have influenced responses and group dynamics. Some young people may not have wanted to disagree with their peers, and thus responded in a similar way to the rest of the group. Due in part to the multi-phase design of the project and the nature of working with youth organizations and schools, we encountered a loss of participants to scheduling conflicts and changes within the youth cohort. Though losing participants is never desirable, it offered valuable lessons on planning research with young people around school and extracurricular schedules, as well as the benefits and challenges of working with group representatives rather than communicating directly with participants. Most participants were non-smokers/vapers, potentially impacting generalizability of the result; in fact, young people who vape have the potential to be exposed to more e-cigarette content and marketing, particularly online (Pettigrew *et al*., 2023). Our use of photos produced by the young people themselves as discussion prompts allowed for participation from people of all groups, and we did not find that non-smokers and non-vapers contributed less to discussions at any stage of the project. If we had not used visual prompts and focused on participants recalling from memory, this may have impacted non-smokers and non-vapers contribution to discussions. Future studies could benefit from focusing on recruiting more young people with personal experiences of vaping. The researchers who conducted data collection are all women of a similar age, which may have impacted on participants’ level of comfort engaging with the activities and sharing their thoughts. We were careful not to respond positively or negatively to participants’ contributions, rather encouraging them to offer more detail on their views whenever possible. As we are public health researchers, it is possible that the young people were reluctant to share positive thoughts or experiences around vaping, which we did our best to mitigate by establishing safe space rules at the outset of all activities.

Youth vaping is a concern, and there is a growing need for comprehensive strategic plans to curtail it. The results from this study should provide guidance for future research to better understand what regulations would be most effective at limiting the appeal of e-cigarette adverts to young people. Exploration of the use of more age-neutral marketing strategies (i.e. restricting the use of bright colours, imagery) and inclusion of warning statements on e-cigarette adverts and the impact this has on youth perceptions would be an important direction to expand this work.

Our findings underscore a public health concern amid the rapid rise of e-cigarettes and evidence linking marketing strategies to increased youth vaping. While progress has been made in regulating e-cigarettes to protect youth—for example, policymakers in the UK and Scotland are considering new regulations including banning disposable e-cigarettes and restrictions on flavours to help tackle the rise in youth vaping—additional restrictions could further reduce exposure and harms for young people (Scottish Government, 2024; UK Government, 2024). Given the findings presented by our study, there is a growing need for policymakers to implement further restrictions to protect young peoples and to restrict the ability of marketers to reach them through enticing adverts.

## CONCLUSION

Our findings highlight that young people feel overly exposed to e-cigarette advertising and they identified aspects of these adverts (including the use of vibrant colours, flavour variations and location of adverts) that they felt were designed to appeal specifically to young people. These findings suggest the need for stronger legislation to protect young people from the advertising and marketing of e-cigarettes. Further research might also contribute to understanding counterarguments and marketing from public health advocates to limit the appeal of e-cigarettes to young people.

## Supplementary Material

daae097_suppl_Supplementary_Appendix_A

daae097_suppl_Supplementary_Appendix_B

daae097_suppl_Supplementary_Appendix_C

daae097_suppl_Supplementary_Appendix_D

daae097_suppl_Supplementary_Appendix_E

## Data Availability

All data relevant to the study are included in the article or uploaded as Supplementary Data.
