# Correction to: Co-production of a youth advocacy video on the harms of e-cigarette advertising in Scotland

**DOI:** 10.1093/heapro/daaf050

**Published:** 2025-04-11

This is a **correction** to:

Marissa J Smith, Caroline Vaczy, Shona Hilton, Co-production of a youth advocacy video on the harms of e-cigarette advertising in Scotland, *Health Promotion International*, Volume 40, Issue 2, April 2025, daae097, https://doi.org/10.1093/heapro/daae097

In the originally published version of this manuscript, the affiliations of authors Marissa J. Smith and Caroline Vaczy were incorrect. The correct affiliations have been added.

