# Supplementary material for: Co-production of a youth advocacy video on the harms of e-cigarette advertising in Scotland
Source: Health Promot Int. 2025 Mar 5;40(2):daae097. doi: 10.1093/heapro/daae097 (PMC11879641; doi:10.1093/heapro/daae097)
Supplement: daae097_suppl_Supplementary_Appendix_A [file daae097_suppl_supplementary_appendix_a.docx]

**Appendix A: Workshop activities and questions**

Workshops were structured around a series of activities in which participants wrote short responses to questions relating to e-cigarette advertising and video development and video development on sticky notes which they placed in different categories. Questions were developed by the research team based on the research questions and information requested by the video production company.

Board 1- What have you seen?

 Q1. Where and how often do you see e-cigarette adverts/displays day to day?

 Q2. How do adverts/displays of e-cigarettes (including online) influence your use of these products?

 Q3. Do you think people choose specific e-cigarette products (e.g. disposables, certain flavours) based on adverts/displays?

 Board 2- What do e-cigarettes do to health?

 Q1. What do you think about the potential health impacts of e-cigarettes?

 Q2. How does this affect your choices to use/not use e-cigarettes?

 Board 3- What should change?

 Q1. How would young people’s purchase/use of e-cigarettes be different if they could not be advertised on social media or in shops?

 Q2 Should the government intervene to protect young people from e-cigarette advertising? Why or why not?

 Q3. If you were a politician/decision-maker, what other rules or steps could be taken to change the e-cigarette marketing and promotions you see?
