# Supplementary material for: Co-production of a youth advocacy video on the harms of e-cigarette advertising in Scotland
Source: Health Promot Int. 2025 Mar 5;40(2):daae097. doi: 10.1093/heapro/daae097 (PMC11879641; doi:10.1093/heapro/daae097)
Supplement: daae097_suppl_Supplementary_Appendix_B [file daae097_suppl_supplementary_appendix_b.docx]

**Appendix B: Focus group coding framework**

| **Themes** | **Subtheme** | **Description** |
| --- | --- | --- |
| Celebrities and influencers advertising e-cigarettes | Celebrities and influencers advertising e-cigarettes | Discussions about why celebrities and influencers would want to advertise e-cigarettes |
|  | Companies using celebrities and influencers | Discussions about why companies use celebrities/influencers to advertise their products |
|  | Inclined to purchase a product | Discussions of whether a product was being advertised/promoted by a celebrity/influencer would influence purchase choices |
| E-cigarette advertising | Design of advert | Discussions of the design of adverts (including colour and text) |
|  | Frequency and location of e-cigarette adverts | Discussions of where young people and how often young people see e-cigarette adverts |
|  | How should e-cigarettes be advertised | Discussions of how e-cigarettes should be advertised |
|  | Influence choice | Discussions of e-cigarette adverts influencing choices |
|  | Recognition of advertising | Do you think these images are advertising a product? |
|  | Recognition of what is being advertised | Discussions of young people being able to recognise advertising |
|  | Social media | Discussions about e-cigarette adverts on social media |
|  | Successful in selling a product | Discussions of the success of adverts in selling a product |
|  | Target audience | Discussion of who young people think adverts are targeted at |
| E-cigarette promotions | Influence choice | Discussions about promotions influencing choices |
|  | Location of promotion | Discussions about where youths see promotions |
|  | Purpose of promotion | What do you think are the purpose of e-cigarette promotions? |
|  | Restriction on e-cigarette promotions | Do you think these promotions are successful in selling a product? |
|  | Successful in selling a product | Discussions about promotions influencing choices |
| Other e-cigarette related thoughts |  | Any other e-cigarette related thoughts |
