# Supplementary material for: Co-production of a youth advocacy video on the harms of e-cigarette advertising in Scotland
Source: Health Promot Int. 2025 Mar 5;40(2):daae097. doi: 10.1093/heapro/daae097 (PMC11879641; doi:10.1093/heapro/daae097)
Supplement: daae097_suppl_Supplementary_Appendix_C [file daae097_suppl_supplementary_appendix_c.docx]

**Appendix C: Video production results**

In the workshops held in Stage 1, in addition to asking the 33 young people about their experiences with and views on the marketing of unhealthy commodities, the researchers collected their thoughts and opinions on the appearance and content of advocacy videos similar to the one we planned to produce. Some questions had unanimous or near-unanimous agreement while others resulted in a more even split of opinions. Views also varied widely between workshop groups.

In each of the sessions on video production, conversations centred on video styles, colours, characters, and content, each of which will be further described in this section. Any quotes are taken from the first workshop activity in which participants wrote down their reactions to each video. The second workshop on styles was not recorded and opinions of the participants were noted down by a member of the research team.

**Styles**

Some video styles were preferred by those with an interest or connection to them. For example, one video that took on the appearance of a video game was polarising, with young people who enjoy video games voting in favour and those who do not voting against.

*“It’s different and unique, relatable to some”*

*“Don’t like the idea of lives”*

While there was no consensus around specific stylistic preferences, the young people did tend to prefer videos that were clear and simple without being childish.

*“The animation was simplistic and […]--- in a non-childish way”*

*“NO! It looked bad, too much changing, very confusing, missed the message”*

Participants expressed concern as to whether the video would be taken seriously, and wanted to choose a video style that would reflect the seriousness of the topic.

*“Animation was funny looking and wouldn’t be taken seriously”*

The young people also tended to prefer videos that would grab and keep people’s attention, engaging them in the video. They did not like videos that seemed ‘boring’.

*“A boring art style”*

*“The animation style created visual interest which made me pay more attention to the video itself”*

**Colours**

As with animation styles, the young people showed an overall preference for attention-grabbing colours that did not seem too childish. Consequently, they generally did not enjoy muted, dark or pastel colours.

*“Yes, they were eye-catching. When things were bad it was duller. You could tell what was good and bad. There was contrast”*

*“Quite dull, made the teachers look like mean people by using dull colours”*

The young people were interested in a colour palette that would help communicate the message and tone of the video, and appreciated the example videos that achieved this.

*“The colours were very representative of the tone of the video”*

*“The colours were simple and didn’t take away from the seriousness of the topic”*

**Content**

The content of the video was based on the young people’s thoughts and experiences around unhealthy food and drink marketing, which is discussed in the sections above. In regards to the video style as it relates to the content, the participants expressed a preference for clarity and storytelling to convey the emotions and experiences of the characters.

*“Not sure about topic”*

*“It helped represent the struggles those with asthma experience by showing her day-to-day routine”*

**Final storyboard workshops**

After conducting the workshops, photo elicitation, and focus group discussions, the research team worked closely with the media production company to translate the young people’s ideas and photos into a script and video storyboard. An additional workshop was then held with four participants from Group 2 with the aim of collecting their feedback and deciding on a final vision for the video. As in the Stage 1 workshops, this session was not recorded but participants’ contributions were collected in notes by the research team.

The media company produced the final video, which was reviewed by the research team and edited based on their feedback. The video was launched on 12th December 2023 in a webinar and can be accessed here: <https://www.youtube.com/watch?v=xwZ-aoQIwjs&t=1s>
