# Supplementary material for: Co-production of a youth advocacy video on the harms of e-cigarette advertising in Scotland
Source: Health Promot Int. 2025 Mar 5;40(2):daae097. doi: 10.1093/heapro/daae097 (PMC11879641; doi:10.1093/heapro/daae097)
Supplement: daae097_suppl_Supplementary_Appendix_D [file daae097_suppl_supplementary_appendix_d.docx]

**Appendix D: Workshop composition**

Table 1 describes the workshop composition. Deprivation rank was assigned using the Scottish Index of Multiple Deprivation (Scottish Government, 2020).

| **Group** | **Area** | **Sex** | **Age** | **Cigarette use** | **E-cigarette use** |
| --- | --- | --- | --- | --- | --- |
| 1 | Most deprived (7)  Least deprived (5) | Mixed: male (1)/ female (11) | 12-16 | Mixed – Never (11)/  Tried (1) | Mixed – Never (8) / Tried (4) |
| 2 | Most deprived (1)  Least deprived (10) | Mixed: male (6)/ female (5) | 14-16 | Mixed – Never (10)/  Tried (1) | Mixed – Never (8) / Tried (3) |
| 3 | Most deprived (9)  Least deprived (1) | Mixed: male (6)/ female (4) | 14-15 | Never | Never |

Table 1: Stage 1 workshop location, composition and participant details.

**References**

SCOTTISH GOVERNMENT. 2020. *The Scottish Index of Multiple Deprivation 2020* [Online]. Available: <https://www.gov.scot/collections/scottish-index-of-multiple-deprivation-2020/> [Accessed 20 December 2023].
