# Supplementary material for: Co-production of a youth advocacy video on the harms of e-cigarette advertising in Scotland
Source: Health Promot Int. 2025 Mar 5;40(2):daae097. doi: 10.1093/heapro/daae097 (PMC11879641; doi:10.1093/heapro/daae097)
Supplement: daae097_suppl_Supplementary_Appendix_E [file daae097_suppl_supplementary_appendix_e.docx]

**Appendix E: Focus group composition**

Table 1 describes the focus group composition. Deprivation rank was assigned using the SIMD (Scottish Government, 2020).

| **Group** | **Area** | **Sex** | **Age** | **Cigarette Smoker** | **E-cigarette use** |
| --- | --- | --- | --- | --- | --- |
| 1A | Most deprived (2)  Least deprived (2) | Female (4) | 12-15 | Never | Mixed – Never (1) / Tried (3) |
| 1B | Most deprived (1)  Least deprived (2) | Female (3) | 13-16 | Mixed – Never (2)/  Tried (1) | Mixed – Never (2) / Tried (1) |
| 2A | Least deprived (5) | Mixed: male (3)/ female (2) | 14-16 | Mixed – Never (4)/  Tried (1) | Mixed – Never (3) / Tried (1) |
| 2B | Most deprived (5)  Least deprived (1) | Mixed: male (3)/ female (3) | 14-16 | Never | Mixed – Never (4) / Tried (2)/ |
| 3A | Most deprived (5) | Mixed: male (4)/ female (1) | 14-15 | Never | Never |
| 3B | Most deprived (4)  Least deprived (1) | Mixed: male (2)/ female (3) | 14-15 | Never | Never |

Table 1: Stage 3 focus group location, composition and participant details.

**References**

SCOTTISH GOVERNMENT. 2020. *The Scottish Index of Multiple Deprivation 2020* [Online]. Available: <https://www.gov.scot/collections/scottish-index-of-multiple-deprivation-2020/> [Accessed 20 December 2023].
